# Myxedema Coma Associated with Macroprolactinoma: Case Report and Review of the Literature

**DOI:** 10.1155/2022/1591616

**Published:** 2022-04-28

**Authors:** Elizabeth Jasola Omoniyi, Richard J. Robbins

**Affiliations:** ^1^From the Division of Endocrinology, Diabetes and Metabolism, Department of Medicine, Houston Methodist Hospital, Houston, TX, USA; ^2^Weill Cornell Medical College, New York, NY, USA

## Abstract

Myxedema coma is a rare life-threatening presentation of severe hypothyroidism associated with a high mortality rate. Although most cases are due to primary thyroid failure, a minority have central hypothyroidism as the underlying cause. We report the case of a 69-year-old man who presented with obtundation, hypoglycemia, and hyponatremia. The patient's initial thyroid-stimulating hormone (TSH) was within normal limits. Subsequent evaluation revealed critical anterior pituitary insufficiency due to a macroprolactinoma and a diagnosis of myxedema coma after appropriate workup The finding of a normal serum TSH should not eliminate the possibility of myxedema coma.

## 1. Introduction

We report a very unusual case of myxedema coma (MC). Severe hypothyroidism meeting the definition of MC is associated with a significant mortality rate (30–50%), even with standard therapy [1, 2]. The underlying causes of myxedema coma vary but usually involve a primary pathological process in the thyroid gland. Commonly, a patient with primary hypothyroidism either stops taking or stops absorbing their thyroid supplements for some time, resulting in fatigue and confusion. Mental status can gradually deteriorate; however, if precipitated by a severe infection, trauma, central nervous system depressants, or a sudden cardiovascular event, rapid onset of stupor or obtundation may ensue, termed myxedema coma. MC has also been reported rarely in patients with central hypothyroidism due to hypothalamic or pituitary diseases. We report a case of myxedema coma with central hypothyroidism associated with a macroprolactinoma.

## 2. Case Report

Following a rare series of three winter storms affecting Houston, TX, at the end of February 2021, there was widespread loss of power and water, and daily temperatures stayed below 0 degrees Celsius for 17 days. An estimated 250–700 Texans died from cold exposure during this period.

**Figure 1:**
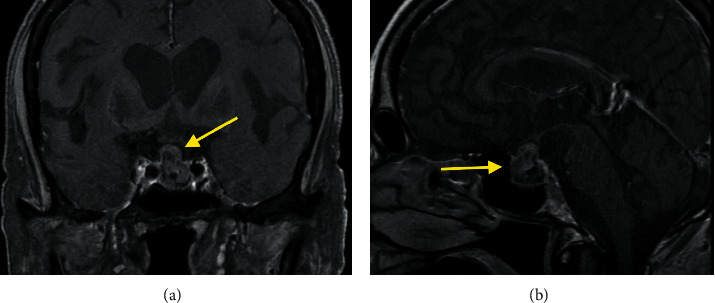
(a) Coronal and (b) sagittal views of the 2 cm pituitary adenoma.

A 69-year-old male was brought to the emergency department in early March 2021 for evaluation of deteriorating mental status leading to disorientation and unresponsiveness. Initial laboratory tests revealed a blood glucose of 45 mg/dl and a serum sodium of 120 mEq/L. His lactic acid level was elevated at 4.2 mmol/L. He was started on intravenous 10% dextrose with no improvement of mental status, though his serum glucose rose to 102 mg/dl. A chest CT revealed a large area of infiltration in the right upper lung consistent with pneumonia. A CT scan of the head revealed calcification of the basal ganglia, thalamus, and cerebral hemispheres bilaterally. A 2 cm mass arising from the sella turcica was also noted (Figure 1). Additional laboratory studies on the day of admission (when he was hypoglycemic) revealed a serum insulin of 1.4 mU/L with normal C-peptide, IGF-2, and proinsulin levels.

He also had a significant medical history of hypertension and iron deficiency anemia that required frequent erythropoietin injections. He also had a history of daily tobacco use but had discontinued that two years prior. There was no history of illicit drug use. His only home medication was lisinopril 20 mg daily. His family reported that he had been increasingly confused and lethargic for one week prior to his emergency room presentation. His family also reported that he had not complained of headaches, nausea, vomiting, or any visual impairment.

Clinically, he was obtunded and only partially responsive to auditory stimuli. His skin was very dry and flaking off; he had generalized edema and macroglossia, but he did not have expressible galactorrhea or gynecomastia. He was variably hypothermic, with body temperature ranging from 94.8–96.1 degrees Fahrenheit. His heart rate ranged from 75 to 86 bpm. An ECG revealed a first-degree AV block. On 2 liters of nasal oxygen, his arterial pO2 was 95 mm·Hg, and his pCO2 was elevated at 48 mm·Hg. In previous medical visits, he had never been diagnosed with thyroid dysfunction, hyponatremia, or hypoglycemia. He had no prior brain imaging.

Given the newly found pituitary lesion and the patient's clinical presentation, pituitary function labs were ordered for further evaluation. His prolactin level was 1,033 ng/ml (4–15 ng/ml); free T4 was 0.2 ng/dl (0.9–1.7 ng/dl); total T3 was low at 35 ng/dl (80–200 ng/dl) with an inappropriately normal thyroid-stimulating hormone (TSH) of 2.35 uIU/ml (0.27–4.2 uIU/ml). His initial (2 PM) serum cortisol was 5 ug/dl with an ACTH of 9.6 pg/ml (7.2–63.3 pg/ml). Following intravenous cosyntropin, his cortisol rose to 10 ug/dl at 30 minutes and 11 ug/dl at 60 minutes. There was no notable change in his mental status after the cosyntropin administration. His IGF-2 was normal at 206 ng/ml (180–580 ng/ml). His initial serum parathyroid hormone level was low at 9 pg/ml.

The patient's serum follicle-stimulating hormone (FSH) was 1.0 mIU/mL (1.6–8.0 mIU/ml), and his serum luteinizing hormone (LH) was 0.3 mIU/ml (1.6–15.2 mIU/ml). An MRI of the brain confirmed a sellar and suprasellar enhancing mass, likely a pituitary adenoma, measuring 1.2 × 1.8 × 2.3 cm, with no sphenoid or cavernous sinus invasion.

The patient's total score of 85 on the widely accepted Popoveniuc et al. diagnostic scoring system [3] confirmed the diagnosis of myxedema coma. He would have received 7 points on Chiong's screening tool [4], making myxedema coma “Likely.” His clinical presentation, history, and multiple laboratory and imaging findings were consistent with myxedema coma and hypopituitarism, likely secondary to a macroprolactinoma.

## 3. Discussion and Literature Review

We report the case of a man who had underlying central hypopituitarism, including partial TSH deficiency, as the cause of his hypothyroidism. He then developed myxedema coma, possibly due to a combination of cold exposure and pneumonia. The cause of his hypopituitarism was most likely a macroprolactinoma. As far as we can determine, this is the first documented case of myxedema coma due in part to a macroprolactinoma. We found no other case reports of this phenomenon in our literature review.

There are many potential underlying causes of MC. Two of the most common are autoimmune thyroid failure (e.g., Hashimoto thyroiditis) and post-thyroidectomy hypothyroidism. Reports of MC due to hypopituitarism with secondary hypothyroidism are quite rare and have been estimated to account for much less than 5% of all MC cases [2]. The earliest case of MC due to central hypothyroidism that we found in our literature review was reported in 1940 by Means, Hertz, and Herman, who reported MC due to Sheehan's disease hypopituitarism. An autopsy revealed atrophy of the pituitary, thyroid, adrenals, and ovaries [5]. In a 1991 literature review, Pinchera et al. estimated that hypothyroidism arising solely from hypopituitarism accounted for approximately 0.005% of all cases of hypothyroidism [6].

The most common precipitating factors in MC are hypothermia, cessation of thyroid supplements, severe infections, anesthetics, tranquilizers, respiratory failure, and stroke [1, 7]. In 2008, Dutta et al. reported on 29 cases of MC. Four (17%) likely had underlying central hypothyroidism, three of which were due to Sheehan syndrome and the last of which was due to a nonfunctioning pituitary adenoma. The precipitating cause of MC was pneumonia in three of the four patients and cold exposure in the fourth patient [8].

Several well-documented cases of MC associated with hypopituitarism have been reported in PubMed over the past 60 years [9–16]. A case with both Hashimoto thyroiditis and Sheehan syndrome leading to MC has also been reported [17], as has a case with Hashimoto thyroiditis and TSH deficiency [18]. In pediatric medicine, Thompson and Henry reported a rare case of MC due to central hypothyroidism in a 7-year-old boy with a chromosome 1q deletion [19].

Given that the vast majority (>95%) of MC cases are due to primary hypothyroidism, it can be difficult to diagnose MC when TSH is not elevated. In patients with MC due to central hypothyroidism, the TSH is typically low or normal, and these patients often have other features of hypopituitarism [8]. Additionally, given that a small but significant percentage of thyroid hormone production from the thyroid gland is TSH-independent (10–15%), these patients often also present with free T4 levels that are higher than in patients with primary hypothyroidism [8].

Although patients with MC due to central hypothyroidism typically present with altered mental status as the first symptom, their neurological impairments may be more subtle than MC due to primary hypothyroidism, in part because there is some minimal TSH-independent T4 production in central hypothyroidism. However, the mortality rate for MC appears to be similar in both central and primary hypothyroidism [8, 20].

Notably, patients with macroprolactinomas have various patterns of hypopituitarism. In a large retrospective review of men with macroprolactinomas (between 20–39 mm in size), 93% had central hypogonadism, 7% had central hypoadrenalism, and 18% had central hypothyroidism [21, 22]. Hyperprolactinemia is commonly associated with diminished or absent pulsatile LH and FSH secretion, likely due to endocrine inhibition of GnRH pulses [23]; however, there is no evidence that it suppresses TSH secretion in men. By contrast, dopamine agonists, which are often used to treat hyperprolactinemia, have been shown to suppress TSH [24].

Regardless of the etiology of the MC, management with high-dose intravenous levothyroxine as a loading dose, followed by a weight-based daily dose, remains the gold standard for therapy to help reverse the severe hypometabolic state in MC patients. Intravenous hydrocortisone should also be administered initially or concurrently, as patients with MC often have relative adrenocortical deficiency, which can worsen as the replacement thyroid hormone increases the metabolic rate [1].

## 4. Conclusion

Myxedema coma is an endocrine emergency that requires a high index of suspicion given its high mortality rate. Unusual underlying causes, such as tumoral hypopituitarism, can impede diagnosis due to the lack of elevated TSH levels. This can potentially delay time-sensitive management therapies. From our literature review and to the best of our knowledge, this is the first report of macroprolactinoma associated with MC and is worth noting, as it highlights the extensive range of complications that can arise from pituitary tumors. Overall, this case highlights the critical importance of careful and thorough clinical assessment in patients with seemingly incongruent clinical and laboratory findings.

## Data Availability

The data used to support the findings of this study are included within the article.
